# Retrospective evaluation of correlation of ABO blood groups among patients with head and neck cancer

**DOI:** 10.6026/973206300213181

**Published:** 2025-09-30

**Authors:** Santoshkumar Kotnoor, Shweta Hegde, Shubhra Raghav, Abida Khatun, Paridhi Sharma, Shobhit K. Saxena, Miral Mehta, Jugajyoti Pathi

**Affiliations:** 1 Department of Oral Pathology and Microbiology, HKE's Society S Nigligappa Dental College and Research, Kalaburagi, Karnataka, India; 2 Department of Oral Medicine and Radiology, Triveni Institute of Dental Science Hospital and Research Centre, Bilaspur, Chhattisgarh, India; 3 Department of Public Health Dentistry, Kothiwal Dental College & Research Centre, Moradabad, Uttar Pradesh, India; 4 Department of Orthodontics and Dentofacial Orthopaedics, Narsinhbhai Patel Dental College and Hospital, Sankalchand Patel University, Visnagar, Gujarat, India; 5 Department of Pediatric and Preventive Dentistry, Karnavati School of Dentistry, Karnavati University, Gandhinagar, Gujarat, India; 6 Department of Oral & Maxillofacial Surgery, Kalinga Institute of Dental Sciences, KIIT Deemed to be University. Bhubaneswar, Odisha, India

**Keywords:** Head and neck cancer, ABO blood group, oral cavity cancer, genetic susceptibility, risk factors

## Abstract

Head and neck cancer (HNC) is a multifactorial disease influenced by genetic and environmental factors. This retrospective case-control study examined the relationship between ABO blood groups and the risk of HNC in 542 patients and 1084 controls. Blood group A was significantly associated with increased HNC risk (OR = 1.42, p = 0.004), particularly for oral cavity cancers, while blood group O showed a borderline protective effect. No significant associations were noted for blood groups B or AB. Thus, we show ABO blood group may play a role in HNC susceptibility.

## Background:

Head and neck cancers (HNC), which include malignancies originating in the oral cavity, oropharynx, hypopharynx and larynx, rank as the seventh most prevalent cancer worldwide, accounting for nearly 900,000 new cases and 450,000 fatalities each year [1]. The principal etiological variables comprise tobacco use, alcohol intake and infection with high-risk human papillomavirus (HPV), especially for oropharyngeal malignancies [2]. Nevertheless, not all individuals exposed to these carcinogens develop head and neck cancer, underscoring the significance of individual vulnerability, including genetic predisposition [3]. The ABO blood group system, defined by distinct carbohydrate antigens (A, B, H) present on red blood cells and different epithelial cells, is among the most extensively researched genetic polymorphisms concerning disease risk [4]. In addition to its essential function in transfusion medicine, research indicates that ABO blood groups affect the risk of several malignancies, such as gastric, pancreatic, and colorectal tumors [5]. Proposed processes encompass the control of cell adhesion, membrane signaling, inflammation, host immunological response and interactions with pathogens [6, 7]. Blood group antigens may act as receptors for certain pathogens or affect the glycosylation patterns of proteins implicated in carcinogenesis and metastasis [8]. Although correlations between ABO blood groups and other cancers are becoming acknowledged, the evidence specifically regarding head and neck cancer (HNC) remains sparse and sometimes inconsistent [9]. Certain studies indicate a heightened risk linked to blood group A [10, 11], but others have noted connections with non-O blood groups in general or identified no significant correlation [12, 13]. A recent meta-analysis suggested a potential moderate elevation in head and neck cancer risk for those with blood group A, although it emphasized considerable heterogeneity across studies and advocated for bigger, rigorously planned research [14]. Therefore, it is of interest to assess the relationship between ABO blood types and the risk of developing head and neck cancer (HNC) in a clearly characterized patient cohort, while also investigating potential variations among major anatomical subsites.

## Materials and Methods:

## Study design and setting:

A retrospective case-control study was conducted at the Department of Otolaryngology-Head and Neck Surgery and the Department of Oncology of a tertiary care teaching hospital.

## Study population:

[1] Cases: Patients aged 18 years or older, with a histologically confirmed primary diagnosis of squamous cell carcinoma (SCC) of the head and neck region (oral cavity, oropharynx, hypopharynx, or larynx) between January 2018 and December 2022, were identified from the hospital's cancer registry and electronic medical records (EMR). Patients with recurrent HNC, metastatic disease from a non-head and neck primary site, history of other malignancies, or incomplete ABO blood group records were excluded.

[2] Controls: Age (± 5 years) and sex-matched individuals without any history of cancer were selected from the hospital's general health check-up database and blood donor registry during the same period. Controls were excluded if they had a personal history of any malignancy or incomplete ABO blood group records. A ratio of 1:2 (cases: controls) was targeted to enhance statistical power.

## Data collection:

Data were extracted from EMRs and archived paper records using a standardized data collection form. For cases, the following information was recorded: demographic details (age, sex), ABO blood group (as documented in pre-transfusion testing records or routine admission workup), primary tumor site (oral cavity, oropharynx, hypopharynx and larynx), tumor stage (AJCC 8th edition), tobacco use history (never, former, current), alcohol consumption history (never, former, current) and HPV status (for oropharyngeal cancers, determined by p16 immunohistochemistry). For controls, demographic details and ABO blood group were recorded.

## ABO blood group determination:

ABO blood group status for both cases and controls was determined using standard serological techniques (forward and reverse grouping) performed by the hospital's transfusion medicine laboratory as part of routine clinical care or pre-donation screening. Results were extracted directly from laboratory reports within the EMR.

## Statistical analysis:

Data were analyzed using SPSS software version 28.0 (IBM Corp., Armonk, NY, USA).

## Results:

Demographic and Clinical Characteristics: The study comprised 542 patients with head and neck cancer (cases) and 1084 matched controls. The average age of cases was 58.7 ± 10.2 years, while the average age of controls was 58.9 ± 9.8 years (p = 0.732). Male participants comprised 72.1% (n=391) of cases and 72.0% (n=780) of controls (p = 0.982). The primary tumor locations among patients were: oral cavity (42.4%, n=230), oropharynx (31.0%, n=168), larynx (22.5%, n=122) and hypopharynx (4.1%, n=22). Tobacco consumption was observed in 68.3% (n=370) of patients and 32.1% (n=348) of controls (p < 0.001). Alcohol use was observed in 55.7% (n=302) of patients and 28.8% (n=312) of controls (p < 0.001). HPV status was determined for 156 oropharyngeal cases, of which 62 (39.7%) were p16-positive. The distribution of ABO blood classes among cases and controls is illustrated in Table 1 (see PDF). In HNC patients, blood group O was the most prevalent at 38.2%, followed by A at 35.8%, B at 19.0% and AB at 7.0%. In the control group, the distribution of blood types was O (42.1%), A (31.5%), B (19.7%) and AB (6.7%). The association between ABO blood groups and head and neck cancer (HNC) risk indicated that, relative to blood group O (reference), blood group A was significantly correlated with an elevated risk of HNC in the unadjusted analysis (OR = 1.42, 95% CI: 1.12-1.80, p = 0.004). The link retained statistical significance following adjustments for age, sex, tobacco consumption and alcohol consumption (Adjusted OR = 1.38, 95% CI: 1.08-1.76, p = 0.010). Blood group O had a marginal protective effect in the unadjusted analysis (OR = 0.82, 95% CI: 0.67-1.01, p = 0.061), which was rendered non-significant following adjustment (Adjusted OR = 0.85, 95% CI: 0.69-1.05, p = 0.130). No statistically significant relationships were detected for blood group B (Adjusted OR = 0.96, 95% CI: 0.74-1.25, p = 0.770) or AB (Adjusted OR = 1.05, 95% CI: 0.72-1.53, p = 0.800) in comparison to blood group O (Table 2 - see PDF). Subgroup analysis indicated differences in the correlation between blood group A and the incidence of head and neck cancer among anatomical subsites (Table 3 - see PDF). The most significant correlation was noted for oral cavity cancers (Adjusted OR = 1.58, 95% CI: 1.18-2.12, p = 0.002). A strong correlation was identified for laryngeal malignancies (Adjusted OR = 1.45, 95% CI: 1.01-2.09, p = 0.045). The correlation for oropharyngeal tumors was less robust and did not achieve statistical significance (Adjusted OR = 1.21, 95% CI: 0.85-1.72, p = 0.290). Subsequent examination of oropharyngeal malignancies categorized by HPV status (p16-positive versus p16-negative) revealed no significant changes in the link with blood group A (data not presented).

## Discussion:

This retrospective case-control study examined the correlation between ABO blood groups and the incidence of head and neck cancer. Our principal discovery is a notable, independent correlation between blood group A and an elevated risk of getting head and neck cancer (Adjusted OR = 1.38, 95% CI: 1.08-1.76, p = 0.010). Additionally, subgroup analysis indicated that this connection was especially significant for oral cavity cancers (Adjusted OR = 1.58) and laryngeal cancers (Adjusted OR = 1.45). Blood type O tended to have a protective effect in unadjusted analysis; however, this effect did not last following correction for confounding variables. No notable correlations were detected for blood groups B or AB. Our findings correspond with other prior research indicating an elevated risk of head and neck cancer associated with blood group A. In line with our results, Kakava *et al.* reported that Greek patients with head and neck cancer also showed a significantly higher prevalence of blood group A, strengthening the consistency of this association across different ethnic groups [15]. A study by Jaleel *et al.* revealed that blood group A was substantially more prevalent among laryngeal cancer patients than in the control group [16]. Aggregated data from various studies determined that blood group A was linked to a modestly elevated risk of head and neck cancer (pooled OR = 1.17, 95% CI: 1.06-1.29) [14], corroborating our results. The observed odds ratio of 1.38 in our investigation, although above the meta-analysis estimate, remains within the range shown by individual studies used in that analysis and illustrates the unique attributes of our study population. Several processes support the biological plausibility of a link between ABO blood types and cancer risk, particularly head and neck cancer (HNC) [17]. ABO antigens are present not only on erythrocytes but also on the epithelial cells that line the mouth cavity, throat and larynx [18]. These antigens can affect cell-cell adhesion, membrane communication and host immunological responses [6, 7]. Individuals with blood group A may display modified inflammatory responses or variations in the glycosylation patterns of proteins associated with tumor growth and metastasis, in contrast to those with blood group O [19]. Altered glycosylation might influence the functionality of adhesion molecules such as E-cadherin and integrins, potentially affecting tumor cell invasion and dissemination [20]. Moreover, ABO blood group status is associated with circulating inflammatory markers such as intercellular adhesion molecule-1 (ICAM-1) and tumor necrosis factor-alpha (TNF-α), which are involved in the tumor microenvironment [21]. The pronounced correlation identified for oral cavity and laryngeal malignancies in our study may be attributed to variations in the density or functional significance of ABO antigen expression in these particular epithelial locations relative to the oropharynx. Our analysis did not reveal a significant correlation between blood group A and the risk of oropharyngeal cancer, even when stratified by HPV status. These contrasts with several studies indicating that ABO status may affect HPV-related malignancies [22]. The absence of correlation in our oropharyngeal subgroup may be attributed to the predominant influence of HPV as a carcinogen in this region, possibly eclipsing the impact of ABO status, or to insufficient statistical power within this subgroup. Conversely, the biological interaction between ABO antigens and HPV infection in oropharyngeal carcinogenesis may be intricate and necessitate additional examination.

The somewhat protective tendency noted for blood group O in unadjusted analysis (OR = 0.82, p = 0.061), which diminished post-adjustment, has been documented in other studies [14, 23]. Individuals with blood group O lack A and B antigens, perhaps diminishing vulnerability to specific infections or alter inflammatory pathways in a distinct manner. Nonetheless, the absence of significance following correction in our study indicates that the observed unadjusted correlation was probably confounded by variables such as tobacco and alcohol consumption, which are highly associated with head and neck cancer and may potentially exhibit intricate associations with blood group distribution. The advantages of our investigation encompass a substantial sample size, histologically verified cases, age- and sex-matched controls, comprehensive data on significant confounders (tobacco, alcohol) and the adjustment for these confounders in the analysis. The subgroup analysis by tumor location offers further specificity. Nonetheless, some limits must be recognized. The retrospective design obviously entails concerns of selection and information bias; however, we alleviated this by employing standardized data extraction from electronic medical records and matching controls. Residual confounding from unmeasured variables (e.g., diet, occupational exposures and comprehensive HPV status for all oropharyngeal patients) cannot be completely ruled out. The study population was sourced from a single tertiary center, which may restrict the generalizability to other ethnic or geographic groups. Ultimately, although we accounted for recognized confounders, the observational design prevents a conclusive causal conclusion.

## Conclusion:

Potential association between ABO blood group A and increased susceptibility to head and neck cancer, particularly of the oral cavity and larynx is shown. Blood group O may offer a modest protective trend. ABO typing could be integrated into broader risk assessments alongside established factors, warranting further prospective studies to clarify its clinical relevance.

## References

[1] Sung H (2021). CA Cancer J Clin..

[2] Hashim D (2016). Ann Oncol..

[3] Chu EA, Kim YJ (2008). Otolaryngol Clin North Am..

[4] Jajosky RP (2022). iScience..

[5] Franchini M (2021). Blood Transfus..

[6] Deng J (2017). Oncotarget..

[7] Amundadottir L (2009). Nat Genet..

[8] Wang Z (2012). Int J Mol Sci..

[9] Iodice S (2010). Eur J Cancer..

[10] Alexandra G (2022). Hematol Rep..

[11] Chihara Y (2005). Lab Invest..

[12] Khalili H (2011). Cancer Epidemiol Biomarkers Prev..

[13] Edgren G (2010). Am J Epidemiol..

[14] Sun W (2015). Cancer Epidemiol..

[15] Kakava K (2016). J BUON..

[16] Jaleel BF, Nagarajappa R (2012). Indian J Dent Res..

[17] Verma P (2021). Ann Maxillofac Sur..

[18] Henry S (1995). Vox Sang..

[19] Zhang BL (2014). Asian Pac J Cancer Prev..

[20] Hakomori S (2010). FEBS Lett..

[21] Barbalic M (2010). Hum Mol Genet..

[22] Shi H (2024). BMJ Open..

[23] Risch HA (2013). Am J Epidemiol..

